# Role of vasopressin and terlipressin in refractory shock compared to conventional therapy in the neonatal and pediatric population: a systematic review, meta-analysis, and trial sequential analysis

**DOI:** 10.1186/s13054-016-1589-6

**Published:** 2017-01-05

**Authors:** Reem Masarwa, Gideon Paret, Amichai Perlman, Shimon Reif, Bruria Hirsh Raccah, Ilan Matok

**Affiliations:** 1 Division of Clinical Pharmacy, School of Pharmacy, Faculty of Medicine, The Hebrew University of Jerusalem, Jerusalem, Israel; 2 Department of Pediatrics, Hadassah-Hebrew University Medical Center, Ein-Kerem, Jerusalem Israel; 3 Department of Pediatric Intensive Care Medicine, Safra Children’s Hospital, Chaim Sheba Medical Center, Ramat-Gan, Israel; 4 Sackler Faculty of Medicine, Tel Aviv University, Tel-Aviv, Israel

**Keywords:** Vasopressin (AVP), Terlipressin (TP), Shock, Septic, Refractory, Vasodilatory, Refractory hypotension, Pediatrics

## Abstract

**Background:**

Vasopressin (AVP) and terlipressin (TP) have been used as last-line therapy in refractory shock in children. However, the efficacy and safety profiles of AVP and TP have not been determined in pediatric refractory shock of different origins. We aimed to assess the efficacy and safety of the addition of AVP/TP therapy in pediatric refractory shock of all causes compared to conventional therapy with fluid resuscitation and vasopressor and inotropic therapy.

**Methods:**

We conducted a systematic review, meta-analysis, and trial sequential analysis (TSA) comparing AVP and TP to conventional therapy. MEDLINE, EMBASE, Cochrane Library, and ClinicalTrials.gov were searched up to February 2016. The systematic review included all reports of AVP/TP use in the pediatric population. Reports of clinical trials were pooled using random-effects models and TSA. Main outcomes were mortality and tissue ischemia.

**Results:**

Three randomized controlled trials and five “before-and-after clinical” trials (without comparator) met the inclusion criteria. Among 224 neonates and children (aged 0 to 18 years) with refractory shock, 152 received therapy with AVP or TP. Pooled analyses showed no association between AVP/TP treatment and mortality (relative risk (RR),1.19; 95% confidence interval (CI), 0.71–2.00), length of stay in the pediatric intensive care unit (PICU) (mean difference (MD), –3.58 days; 95% CI, –9.05 to 1.83), and tissue ischemia (RR, 1.48; 95% CI, 0.47–4.62). In TSA, no significant effect on mortality and risk for developing tissue ischemia was observed with AVP/TP therapy.

**Conclusion:**

Our results emphasize the lack of observed benefit for AVP/TP in terms of mortality and length of stay in the PICU, and suggest an increased risk for ischemic events. Our TSA suggests that further large studies are necessary to demonstrate and establish benefits of AVP/TP in children.

PROSPERO registry: CRD42016035872

**Electronic supplementary material:**

The online version of this article (doi:10.1186/s13054-016-1589-6) contains supplementary material, which is available to authorized users.

## Background

Hemodynamic shock is a leading cause of morbidity and mortality in the pediatric population worldwide [1]. A delay in treating shock may result in irreversible organ damage [2, 3]. Morbidity from shock may include renal failure, disseminated intravascular coagulation (DIC), and death [1–3]. Early goal-directed therapy is targeted at maintaining and restoring an adequate ventilation and circulation within the first hour of shock onset [4–6].

Aggressive fluid resuscitation is the first line of therapy for shock [7]. Thereafter, hemodynamic support is achieved with vasopressors and inotropes [5, 7]. However, reduced vasoconstrictor sensitivity to vasopressors in shock can lead to vasodilation, severe hypotension, and vasoparalysis [8]. There is, therefore, a pressing need for agents which target other pathways involved in the development of shock.

In the past decade, arginine-vasopressin (AVP) has emerged as a potentially useful therapy for refractory shock [9]. AVP acts on V1 receptors located on vascular smooth muscle leading to an increase in mean arterial pressure (MAP). Patients with shock exhibit inappropriately low circulating AVP concentrations [7, 8, 10]. From a biologic perspective, the basic rationale behind the addition of AVP/TP in refractory shock is the depletion of endovascular AVP in states of shock [11]. Furthermore, AVP/terlipressin (TP) may contribute to MAP elevation in the refractory shock state since vascular smooth muscle shows a decreased ability to contract, and the hypotension may be refractory to standard catecholamine vasopressor therapy [12].

AVP and TP administration in states of shock may be beneficial in improving cardiovascular parameters, such as MAP and heart rate (HR) [13, 14]. AVP/TP may also be used in vasodilatory shock following cardiopulmonary bypass [15].

There is a paucity of data regarding the outcomes of the use of AVP/TP in refractory shock in children. A Cochrane review from 2013, which aimed to assess the efficacy and safety of AVP in neonates with refractory shock, did not find sufficient evidence to recommend or refute the use of AVP [16]. We therefore sought to conduct a systematic review, meta-analysis, and trial sequential analysis (TSA) to evaluate the efficacy and safety of AVP/TP in critically ill neonates and children with refractory shock of different origins compared to conventional therapy with fluid resuscitation, vasopressor, and inotropic therapy.

## Methods

### Data sources and searches

This systematic review followed the Preferred Reporting Items for Systematic Reviews and Meta-analysis (PRISMA 2009) framework guidelines and the review protocol was registered at the PROSPERO registry of systematic reviews on February 2016 (registry number: CRD 42016035872) [17, 18]. The systematic review was performed using MEDLINE, EMBASE, and Cochrane through February 2016 to identify all published randomized controlled trials (RCTs), prospective clinical trials (“before and after”, without a comparator group), cohort studies, case-control studies, and case series involving the treatment/comparison of AVP or TP (in addition to conventional therapy) in pediatric refractory shock (of septic, vasodilatory, or mixed origin) to conventional therapy. Relevant studies were identified using the following search terms: “vasopressin”, “terlipressin”, “shock”, “septic”, “refractory”, “hypotension”, “vasodilatory”, “mixed”, “children”, “neonates”. We subsequently searched and evaluated published systematic reviews, online resources, conference abstracts, clinicaltrials.gov, and expert opinion to ensure identification of all published and unpublished studies. No language or date restrictions were applied to the search. No approval from the Institutional Review Board was required.

### Study selection and data extraction

The studies were identified through a search by one reviewer (RM), and the abstracts were independently screened by two reviewers (RM and BHR). Disagreements were resolved by consensus and/or referral to a third reviewer (IM). The full text of the resulting references was then retrieved by one reviewer (RM). The primary endpoints of this analysis were mortality outcomes (30-day mortality in RCTs or mortality during/by end of trial in non-RCTs) and tissue ischemia (new onset or worsening of existing condition). Secondary outcomes included hemodynamic indices, vasoactive score, and the length of stay in the pediatric intensive care unit (PICU).

### Selection criteria

We applied the following screening criteria to determine qualitative eligibility for inclusion in the meta-analysis: prospective clinical studies (RCTs and prospective “before and after ” clinical trials, without comparator group) of children aged 0 to 18 years and in refractory shock, which compared AVP/TP (in addition to conventional therapy) to conventional therapy and reported on mortality, morbidity, at least two hemodynamic indices, and adverse effects, and which met 1b and 1c levels of evidence of the Oxford Centre for Evidence-based Medicine (CEBM) levels of evidence 2011 [19]. We excluded studies of AVP/TP for other indications, or with no full-text access. Retrospective and prospective observational studies and case reports were included in the systematic review only.

### Quality assessment and risk of bias

The methodological quality of the studies included was assessed using the CEBM levels of evidence 2011 [19]. Risk of bias was assessed according to the Cochrane Collaboration methodology [20].

### Data synthesis

We conducted a meta-analysis to pool the results of trials comparing conventional therapy only with conventional therapy plus AVP/TP using Comprehensive Meta-analysis (CMA 3.0). For TSA, the TSA program v.0.9 beta was used (http://www.ctu.dk/tsa/downloads.aspx). For bias risk assessment we used Review Manager (RevMan), Version 5.3. Copenhagen (The Nordic Cochrane Centre, The Cochrane Collaboration, 2014). The primary comparison themes were mortality and tissue ischemia. Secondary comparison themes included vasoactive score, the length of stay in the PICU, and hemodynamic measures. For studies reporting only the interquartile range (IQR) for continuous measure outcomes, we assumed that the mean and median were equal, and calculated the standard deviation (SD) from IQR by dividing the IQR by 1.35 [21]. To calculate the change in continuous hemodynamic measures we estimated the standard deviation from the pooled variance of each measure. Heterogeneity was assessed using the *I* ^2^ statistic. Values of 25%, 50%, and 75% for *I* ^2^ represented low, medium, and high heterogeneity, respectively [22]. We used random-effects models to pool results. We calculated risk ratios (RRs) for dichotomous outcomes when including only RCTs in the analysis. Event rates (ERs) were calculated for dichotomous outcomes including RCTs and “before-and-after clinical” trials (without comparator). Event rates are a measure of how often an event occurred in a group [23], and are measures of the occurrence of an event for each participant over the time they were observed. Pooled event rates from the "before and after" clinical trials can provide an estimate of the expected rates of these events in the settings evaluated; however, they do not provide a direct estimate of the relative effect of the intervention compared to a control. The mean difference (MD) was calculated for continuous outcomes, with their corresponding 95% confidence intervals (CIs). Statistical significance was defined using a two-sided α of 0.05, and interpretations of clinical significance emphasized CIs.

### Trial sequential analysis

Meta-analyses may result in type I errors owing to an increased risk of random error when sparse data are analyzed [24]. TSA allows for controlling the *p* value when scarce data exist and clear conclusions cannot be drawn [25, 26]. TSA allows the quantification of the required sample size for determining the effect under study while adjusting the threshold for statistical significance [25, 26]. The threshold for reaching statistical significance adjusts the CIs and reduces type I errors. When the cumulative z-curve crosses the threshold boundaries, one may conclude that a sufficient level of evidence for the intervention effect has been reached and no further trials are needed. If the z-curve does not cross any of the boundaries, evidence to reach a conclusion is insufficient [25]. We used TSA adjusted random-effects models to pool results from RCTs for primary outcomes. We conducted two- and one-sided TSA to maintain a risk of 5% for type I error and a power of 80%. We used the estimated function to calculate the required information size (IS). We calculated 95% CIs adjusted for repetitive testing.

## Results

### Trial flow

**Fig. 1:**
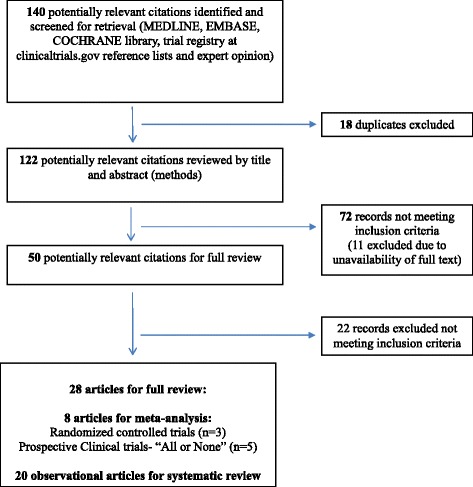
Publication selection and search process

Our search yielded 140 relevant titles. Initial screening led to the exclusion of 18 duplicate records and 72 records that did not meet inclusion criteria. The remaining 50 publications were retrieved for full-text review. Twenty-two publications were excluded based on inclusion criteria, leaving 28 studies. Twenty studies were observational and were included in the systematic review only (Additional file 1: Table S1), leaving eight clinical publications for meta-analysis. The selection process is illustrated in Fig. 1.

### Characteristics and quality of clinical studies included in the meta-analysis

The studies included in the analysis are detailed in Table 1. In total, 224 children received AVP/TP. In all reports, conventional therapy with volume resuscitation and vasopressors/inotropes were given prior to the initiation of AVP/TP (excluding one study which included volume resuscitation or dopamine) [27]. Three RCTs [27–29] and five clinical trials [30–34] met the 1b and 1c level criteria of the CEBM [19], and two RCTs had a low risk for bias (Additional file 2: Figure S1) [27, 29]. The studies included evaluated the effect of AVP/TP in the pediatric population over a relatively wide range of ages, and the majority of the studies were not restricted to shock arising from a specific mechanism.

**Table 1:** Published RCTs and clinical trials on the use of vasopressors in neonates and children sorted by study design (quality of evidence)

Author, year	Study design	Type of shock/indication for AVP/TP administration	Age	Number of subjects by treatment received	Treatment (medication, dose, duration)	Outcomes	Adverse events	Study period	Oxford levels of evidence 2011
Choong et al., 2009 [29]	Randomized controlled double-blind trial (RCT)	Vasodilatory	1 month to 17 years	69 children: 35, AVP; 34, placebo	IV AVP continuous infusion: 0.0005–0.002 units/kg/min	Mortality: AVP, 10/33; placebo, 5/32; *p* = 0.21 MAP: AVP, increase; placebo, increase; *p* = 0.02 HR: NA UO: AVP, decrease; placebo, decrease; *p* = 0.65 Catecholamine requirement: AVP, unchanged; placebo, unchanged; *p* = 0.93 Vasoactive score: AVP,: unchanged; control, unchanged; *p* = 0.93	New onset tachycardia: 0 Tissue ischemia/skin lesions: AVP, 2; placebo, 0; *p* = 0.15 Cardiac arrest: AVP, 1; placebo, 0; *p* = 0.55 Rhabdomyolysis: 0 Metabolic acidosis: 0	4 years	1b
Rios and Kaiser, 2015 [27]	Randomized controlled double-blind trial (RCT)	Refractory hypotension	<24 h (mean age: 6.5 h)	70 ELBW infants: 10, AVP; 10, dopamine; 50, control	IV AVP continuous infusion: 0.0001–0.0006 units/kg/min	Mortality: AVP, 1/10; dopamine, 1/10; control, 0/50; *p* = 0.261 MAP: AVP, increase (9/10); dopamine, increase (8/10); control, NA; *p* = 1.0 HR: AVP, unchanged; dopamine, increase; control, NA; *p* < 0.01 UO: AVP, unchanged; dopamine, unchanged; control, unchanged; *p* = 0.384 Catecholamine requirement: AVP, unchanged; placebo, unchanged; *p* = 0.93 Vasoactive score: NA	New onset tachycardia: 0 Tissue ischemia/skin lesions: AVP, 0: dopamine, 1; control, 2; *p* = 0.15 Cardiac arrest: AVP, 1; placebo, 0; *p* = 0.801 Rhabdomyolysis: NA Metabolic acidosis: NA	2 years	1b
Yildizdas et al., 2008 [28]	Clinical, non-blind, controlled trial,	Septic	1 month to 5.5 years	58 children: 30, TP; 28, control	IV TP bolus: 20 μg/kg every 6 h	Mortality: TP, 20/30; control, 20/28; *p* = 0.1 MAP: TP, increase; *p* = 0.001; control, NA HR: TP, decrease, *p* = 0.001, control, NA UO: TP, unchanged;, control, unchanged; *p* = 0.2 Catecholamine requirement: NA Vasoactive score: NA	New onset tachycardia: 0 Tissue ischemia/skin lesions: TP, 5; control, 3; *p* = 0.3 Cardiac arrest: 0 Rhabdomyolysis: 0 Metabolic acidosis: 0	6 months	1b
Agrawal et al., 2012 [30]	Clinical trial	Vasodilatory (post-cardiac surgery)	1 month to 8.5 years	12 children	IV AVP continuous infusion: 0.0005–0.03 units/kg/min	Mortality: 3/12 Morbidity: NA MAP: increase; *p* < 0.001 HR: unchanged; *p* = 0.188 UO: NA Catecholamine requirement: decrease; *p* < 0.001 Vasoactive score: after AVP, decrease; *p* = 0.001	New onset tachycardia: 0 Tissue ischemia/skin lesions: 0 Cardiac arrest: 0 Rhabdomyolysis: 0 Metabolic acidosis: 0	6 months	1c
Rodriguez-Núñez et al., 2010 [31]	Clinical trial	Septic	24 days to 15 years	15 children	IV TP loading dose: 20 μg/kg continuous infusion: 4–20 μg/kg/h	Mortality: 7/15 Morbidity: NA MAP: increase; *p* < 0.05 HR: decrease; *p* < 0.05 UO: NA Catecholamine requirement: decrease, *p* < 0.05	New onset tachycardia: 0 Tissue ischemia/skin lesions: 4 Cardiac arrest: 1 Rhabdomyolysis: 4 Metabolic acidosis: 3	32 months	1c
Rodriguez-Núñez et al., 2006 [32]	Clinical trial	Septic	1 month to 13 years	16 children	IV TP bolus: 0.02 mg/kg every 4 h, for a maximum of 17 h	Mortality: 9/16 Morbidity: NA MAP: increase; *p* < 0.01 HR: unchanged; *p* = not significant UO: NA Catecholamine requirement: decrease; *p* < 0.05 Vasoactive score: after AVP, decrease; *p* < 0.05	New onset tachycardia: 0 Tissue ischemia/skin lesions: 5 Cardiac arrest: 0 Rhabdomyolysis: 2 Metabolic acidosis: 0	12 months	1c
Bidegain et al., 2010 [34]	Observational-retrospective	Refractory hypotension/septic	1 day to 8 months	20 children	IV AVP continuous infusion: 0.00017–0.0007 units/kg/min	Mortality: 13/20 Morbidity: NA MAP: increase; *p* = 0.002 HR: decrease; *p* = 0.45 UO: decrease; *p* = 0.36 Catecholamine requirement: decrease; dopamine, *p* = 0.006; epinephrine, *p* = 0.04	New onset tachycardia: 0 Tissue ischemia/skin lesions: 0 Cardiac arrest: 0 Rhabdomyolysis: 0 Metabolic acidosis: 0	2.5 years	1c
Matok et al., 2005 [33]	Observational-retrospective	Septic	4 days to 17.7 years	14 children	IV TP: loading dose: 7 μg/kg/dose, twice daily maintenance: 20 μg/kg every 6 h	Mortality: 8/14 Morbidity: NA MAP: increase; *p* = 0.001 HR: decrease; *p* = 0.003 UO: increase, *p* = 0.011 Catecholamine requirement: decrease, 8/14; *p* = NA	New onset tachycardia: 0 Tissue ischemia/skin lesions: 0 Cardiac arrest: 0 Rhabdomyolysis: 0 Metabolic acidosis: 0	1 year	1c

The studies in Table 1 were included in the meta-analysis

Mortality-refers to pediatric/neonatal intensive care unit mortality

*AVP* arginine-vasopressin, *ELBW* extremely low birth weight, *HR* heart rate, *IV* intravenous, *MAP* mean arterial pressure, *NA* not available, *TP* terlipressin, *UO* urine output

### Meta-analysis

#### Mortality

**Fig. 2:**
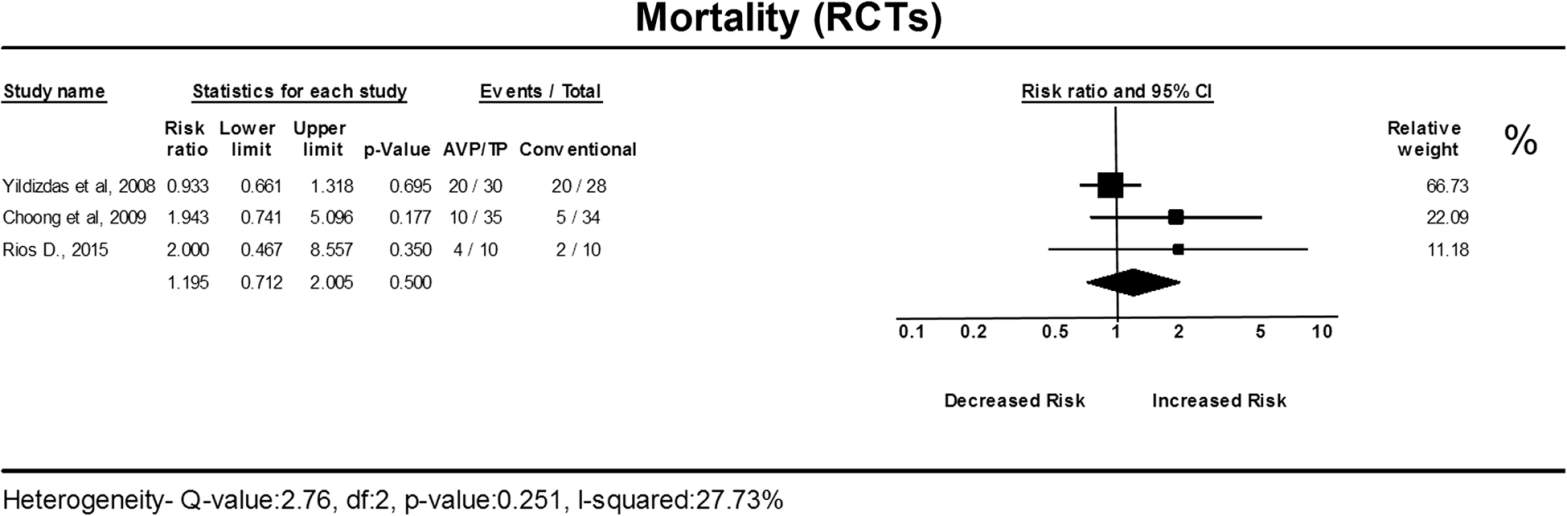
The risk ratio for mortality in randomized controlled trials (*RCTs*). The forest plot demonstrates point estimates of risk ratio surrounded by 95% confidence interval (*CI*). *AVP* arginine-vasopressin, *TP* terlipressin

**Fig. 3:**
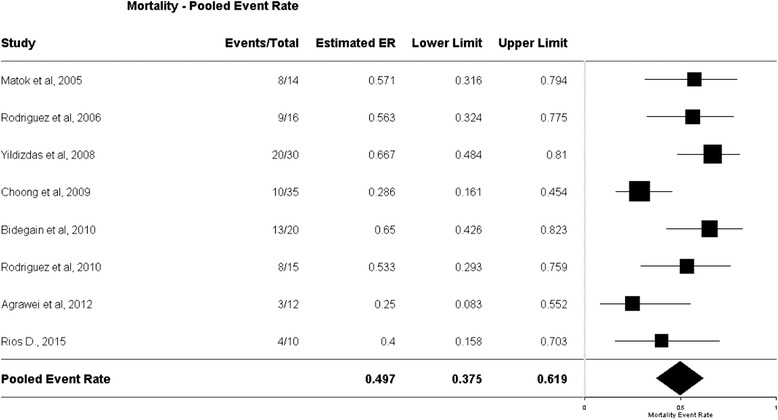
Event rate for mortality in all clinical trials. The plot demonstrates point estimates of event rates surrounded by 95% confidence interval (*CI*). *ER* event rate

**Fig. 4:**
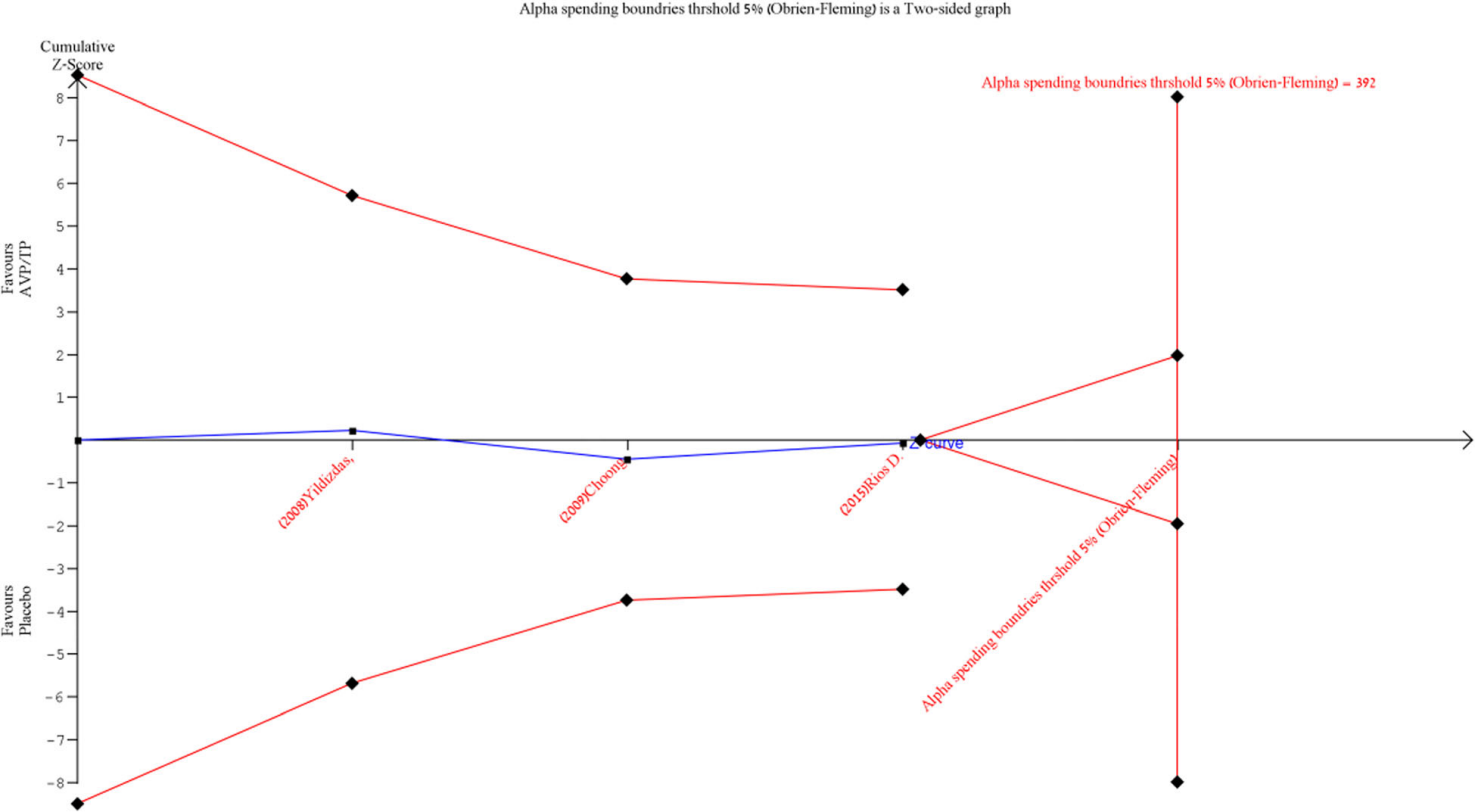
Trial sequential analysis for mortality in randomized controlled trials: a relative risk of 1.01, two-sided boundary, incidence of 14.2% in the control arm, incidence of 25.5% in the treatment arm, a low bias estimated relative risk reduction of 80%, α of 5%, power of 80% were set. There is an estimated required information size of 392 randomized patients that are not reached. The boundaries for futility are not crossed and no effect on mortality is observed; the z-curve is parallel to the boundary of the treatment group. *AVP* arginine-vasopressin, *TP* terlipressin

The addition of AVP/TP to vasopressor/inotropic therapy in refractory shock had no significant effect on mortality. Analysis of RCTs resulted in a non-significant difference in risk compared to conventional treatment, with a RR of 1.19 with low heterogeneity (95% CI, 0.71–2.00; *I* ^2^ = 28%) (Fig. 2). The mortality rate among patients treated with AVP/TP in the clinical trials was high, with a pooled ER of 0.49 (95% CI, 0.37–0.61) (Fig. 3). In TSA, the boundary for futility was not crossed, and no effect on mortality was observed; the estimated IS to reach the futility boundaries was 392 randomized patients (Fig. 4).

#### Tissue ischemia

**Fig. 5:**
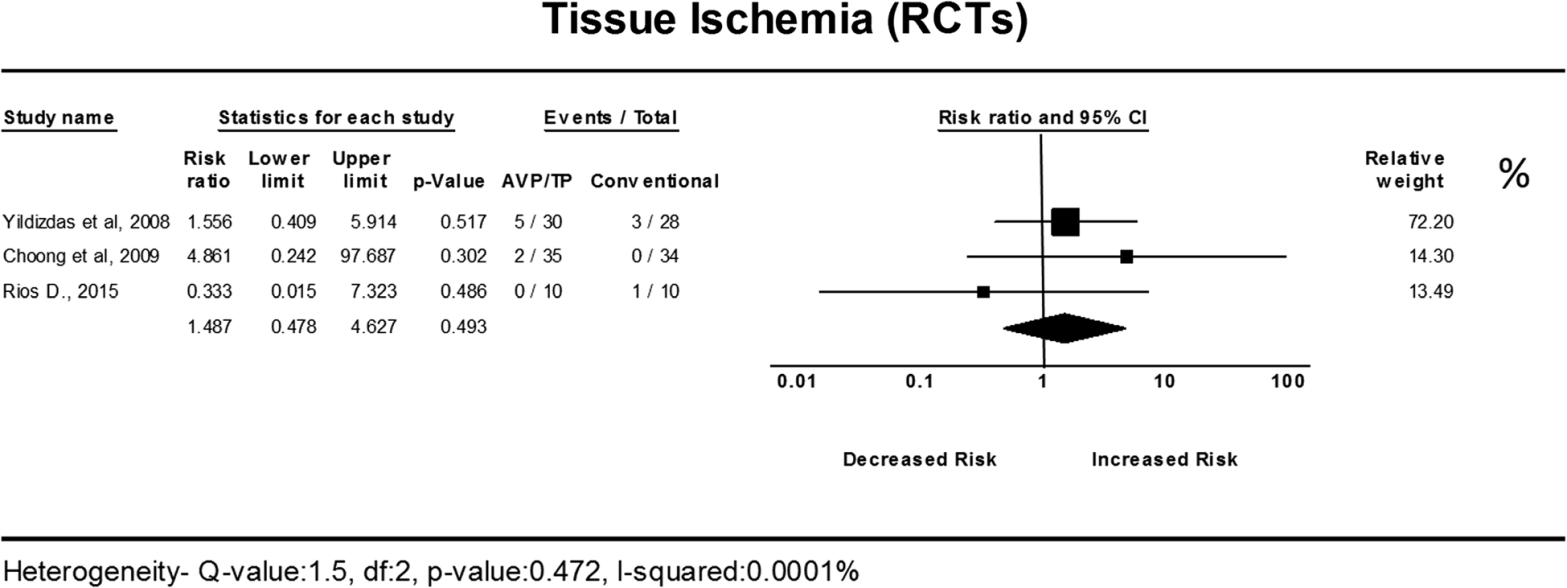
The risk ratio for tissue ischemia in randomized controlled trials (*RCTs*). The forest plot demonstrates point estimates of risk ratio surrounded by 95% confidence interval (*CI*). *AVP* arginine-vasopressin, *TP* terlipressin

The addition of AVP/TP to vasopressor/inotropic therapy in refractory shock had no significant effect on the risk for developing tissue ischemia. Analysis of RCTs resulted in a non-significant difference in risk compared to conventional treatment, with a RR of 1.48 with low heterogeneity (95% CI, 0.47–4.62; *I* ^2^ = 0%) (Fig. 5). The tissue ischemia rate among patients treated with AVP/TP in the clinical trials was considerable, with a pooled ER of 0.16 (95% CI, 0.08–0.28) (Additional file 3: Figure S2). In TSA, the boundary for futility was not crossed, and no effect on tissue ischemia was observed; the estimated IS to reach the futility boundaries was 231 randomized patients (Additional file 4: Figure S3).

#### Vasoactive score

The addition of AVP/TP in refractory shock resulted in a significant decrease in the vasoactive score compared to standard therapy, with a pooled MD of –14.13 units and low heterogeneity (95% CI, –20.61 to –7.66; *I* ^2^ = 38.25%) (Additional file 5: Figure S4).

#### Mean arterial pressure

The addition of AVP/TP in refractory shock resulted in a significant increase in the MAP, with a pooled MD of 12.34 mmHg and high heterogeneity (95% CI, 6.38–18.3; *I* ^2^ = 93%) (Additional file 6: Figure S5).

#### Heart rate

The addition of AVP/TP in refractory shock resulted in a significant decrease in the HR, with a pooled MD of –12.25 beats per minute and intermediate heterogeneity (95% CI, –18.96 to –5.55; *I* ^2^ = 67%) (Additional file 7: Figure S6).

#### Length of stay in the PICU

The addition of AVP/TP in refractory shock did not result in a significant decrease in the length of stay, with a pooled MD of –3.58 days (reduction) and intermediate heterogeneity (95% CI, –9.05 to 1.83; *I* ^2^ = 54%) (Additional file 8: Figure S7).

## Discussion

In this meta-analysis, no significant difference in mortality risk was found between AVP/TP and conventional treatment for refractory shock in the pediatric population. Furthermore, after applying TSA, the non-significant effect on mortality outcomes remained; however, a large number of randomized patients would be required to reach the futility boundary. Although no significant difference in tissue ischemia risk was found, when applying TSA we found a trend towards a higher risk for tissue ischemia.

AVP/TP treatment did significantly improve hemodynamic indices, with a significant reduction in the vasoactive score, HR, and an increase in MAP.

Our meta-analysis indicates that AVP/TP therapy is ineffective in reducing mortality in refractory shock in the pediatric population. Our results demonstrate that mortality rates among patients treated with AVP/TP in the clinical trials were high, as evidenced by the pooled ER estimate for mortality. Moreover, analysis of RCTs resulted in a non-significant difference in the relative risk for death between the treatment and control groups. The high mortality rates in the studies included in our analysis can be attributed to the high risk for mortality characteristic of refractory shock despite proper treatment [4]. However, our results indicate that a very large number of patients would be necessary to reach the information size required to reach the futility boundaries for mortality, as shown by TSA.

AVP acts on V1 receptors located on vascular smooth muscle leading to vasoconstriction, and therefore therapy with AVP/TP may put the patients at a higher risk for developing tissue ischemia [9]. In adults, RCTs showed a trend towards developing tissue ischemia with AVP/TP therapy [35, 36]. Our meta-analysis found high event rates for tissue ischemia among AVP/TP-treated patients.

The vasoactive score has previously been shown to be associated with morbidity and mortality in PICUs [37, 38], and also has been associated with improvement in length of stay in the PICU [38]. Although AVP/TP treatment resulted in lower vasoactive scores, it did not significantly reduce clinically important outcomes such as mortality and the length of stay in the PICU.

Our analysis found a significant increase in MAP and a decrease in HR, in line with previous reports. These effects may be attributed to the mechanism by which AVP/TP causes vasoconstriction, and to the decreased sensitivity of vascular smooth muscle to catecholamines in refractory shock [37–39]. However, this did not seem to translate into a meaningful improvement in clinically significant outcomes.

Unfortunately, most of the studies included in our meta-analysis did not address the mechanism of shock and did not report on performing echocardiography during the study period, and, consequently, may have included patients with different mechanisms of shock. In light of the absence of information regarding ventricular function in most of the studies identified, and the fact that the outcome of neonatal and pediatric shock relies on ventricular function, we were unable to assess the relationship between the mechanism of shock and goal of treatment and the effectiveness of AVP/TP. As AVP and TP do not possess inotropic properties they are far less likely to be of use in states of shock where ventricular dysfunction is present. Additionally, the meta-analysis included studies with children of different ages. As scarce high-quality literature exists regarding the use of AVP/TP in neonates and children with refractory shock, a separate statistical analysis for neonates and children was not feasible. Our results are therefore limited by the nature of the available studies, as there may be difference between neonates and older children, and between different types of shock, regarding the mechanism of shock compensation and drug doses, as well as the potential for adverse effects. For example, the presence of DIC in refractory shock is probably higher than less severe types of shock, and AVP/TP is more likely to provide benefit in vasodilatory shock compared to cardiogenic shock [40].

## Conclusion

The results of our analysis emphasize the lack of observed benefit for AVP/TP in terms of mortality, as well as a possible association with ischemic events. While the data available on this topic are limited, and the possibility of a small benefit cannot be ruled out, our trial sequential analysis suggests that further studies are unlikely to demonstrate an improvement in mortality; however, more studies would be necessary to demonstrate this conclusively. Additionally, further studies of AVP/TP incorporating an evaluation of the mechanism of shock could enhance our understanding of the clinical utility of AVP/TP for specific types of shock.

We believe these findings are quite important, as they can both inform current practitioners as to the best available evidence regarding the efficacy and safety of these agents.

## Additional files

Additional file 1: Table S1. Published observational reports on the use of vasopressors in neonates and children. (DOCX 42 kb)
Additional file 2: Figure S1. Risk of bias summary: review authors' judgements about each risk of bias item for each included study. (DOCX 23 kb)
Additional file 3: Figure S2. Event rate for tissue ischemia in all clinical trials. The plot demonstrates point estimates of event rate surrounded by 95% CI. (DOCX 48 kb)
Additional file 4: Figure S3. Trial sequential analysis for tissue ischemia in randomized controlled trials. A relative risk of 2.72, one-sided lower boundary, an incidence of 1.6% in the control arm, an incidence of 11% in the treatment arm, a low bias estimated relative risk reduction of 85%, α of 5%, power of 80% were set. An estimated required information size of 231 randomized patients was not reached. The boundaries for futility are not crossed and no effect on tissue ischemia is observed. (DOCX 120 kb)
Additional file 5: Figure S4. Mean difference for vasoactive score. The forest plot demonstrates point estimates of mean difference surrounded by 95% CI. (DOCX 138 kb)
Additional file 6: Figure S5. Mean difference for mean arterial pressure. The forest plot demonstrates point estimates of mean difference surrounded by 95% CI. (DOCX 155 kb)
Additional file 7: Figure S6. Mean difference for heart rate. The forest plot demonstrates point estimates of mean difference surrounded by 95% CI. (DOCX 153 kb)
Additional file 8: Figure S7. Mean difference for length of stay in intensive care. The forest plot demonstrates point estimates of mean difference surrounded by 95% CI. (DOCX 133 kb)

## Abbreviations

AVP: Arginine-vasopressin
CEBM: Center for Evidence-based Medicine
CI: Confidence interval
CMA: Comprehensive Meta-analysis
DIC: Disseminated intravascular coagulation
ER: Event rate
HR: Heart rate
IQR: Interquartile range
IS: Information size
MAP: Mean arterial pressure
MD: Mean difference
PICU: Pediatric intensive care unit
RCT: Randomized controlled trial
RR: Risk ratio
TP: Terlipressin
TSA: Trial sequential analysis
