# Supplementary material for: Role of vasopressin and terlipressin in refractory shock compared to conventional therapy in the neonatal and pediatric population: a systematic review, meta-analysis, and trial sequential analysis
Source: Crit Care. 2017 Jan 5;21:1. doi: 10.1186/s13054-016-1589-6 (PMC5217634; doi:10.1186/s13054-016-1589-6)
Supplement: Additional file 1: Table S1. — Published observational reports on the use of vasopressors in neonates and children. (DOCX 42 kb) [file 13054_2016_1589_MOESM1_ESM.docx]

| **Additional file 1: Table S1.** Published observational reports on the use of vasopressors in neonates and children | | | | | | | | | |
| --- | --- | --- | --- | --- | --- | --- | --- | --- | --- |
| Oxford levels of evidence 2011 | Study period | Adverse events (secondary outcome) – number of episodes | Outcome (primary, secondary) | Treatment (medication, dose, duration) | Number of subjects by treatment received | age | Type of shock/indication for AVP/TP administration | Study design | Author, year |
| Oxford-4 | 1 year | New onset tachycardia-0  Tissue ischemia/skin lesions-0  Cardiac arrest-0  Rhabdomyolysis-0  Metabolic acidosis-0 | Mortality-2/3  Morbidity- NA  MAP- 1:increase, 2-3: no change  HR- decrease  UO- 1: increase  Catecholamine requirement-1:unchanged, 2-3:increase | IV AVP:  Neonate 1: 0.025 units-bolus and then continuous infusion: 0.0005 units/kg/minute  Neonates 2 and 3 continuous infusion: 0.0001-0.001 units/kg/minute | 3 neonates (VLBW) | 3 days – 2 weeks | Septic and cardiogenic (1-septic, 2-3- cardiogenic) | Observational-prospective | Meyer et al, 2006[41] |
| Oxford-4 | 1.9 years | New onset tachycardia-0  Tissue ischemia/skin lesions-1  Cardiac arrest-0  Rhabdomyolysis-0  Metabolic acidosis-0 | Mortality- septic:1/3, non-septic: 3/3  Morbidity- NA  MAP- increase: 6/6  HR- septic: decrease: 3/3, non-septic: unchanged: 3/3  UO-NA  Catecholamine requirement: increase: 2/6, unchanged: 4/6 | IV AVP continuous infusion: 0.0005-0.006 units/kg/minute | *6 neonates (ELWB)- 3 neonates were included in previous report | Neonates (days) | Septic (3) and non-septic (3) | Observational-prospective | Meyer et al, 2006[42] |
| Oxford-4 | 4 years | New onset tachycardia-NA  Tissue ischemia/skin lesions-NA  Cardiac arrest-NA  Rhabdomyolysis-NA  Metabolic acidosis-NA | Mortality-4/12  Morbidity- NA  MAP-NA  HR-NA  UO-increase 8/12  Catecholamine requirement-NA | IV AVP continuous infusion:  0.00002-0.00004 units/kg/minute | 12 children | 4 days – 19 years | Hypotensive | Observational-retrospective | Masutani, 2005[43] |
| Oxford-4 | 1 year | New onset tachycardia-0  Tissue ischemia/skin lesions-0  Cardiac arrest-0  Rhabdomyolysis-  Metabolic acidosis-0 | Mortality-7/8  Morbidity- NA  MAP-NA  HR-unchanged  UO- unchanged  Catecholamine requirement- decrease, p<0.05 | IV AVP continuous infusion: 0.0003-0.005 units/kg/minute | 8 children | 1 month – 12 years | Septic/hemorrhagic/low cardiac output | Observational-retrospective | Efrati et al, 2004[44] |
| Oxford-4 | 3.10 years | New onset tachycardia-0  Tissue ischemia/skin lesions-0  Cardiac arrest-0  Rhabdomyolysis-0  Metabolic acidosis-0 | Mortality-7/22  Morbidity- NA  MAP-NA  HR-NA  UO- increase, p<0.0001  Catecholamine requirement-NA | IV AVP continuous infusion: 0.001-0.02 units/kg/minute | 22 neonates | Newborns (2 neonates)- 24 days | Refractory hypotension/septic | Observational-retrospective | Ikegami et al, 2010[45] |
| Oxford-4 | 2.9 years | New onset tachycardia-0  Tissue ischemia/skin lesions-0  Cardiac arrest-  Rhabdomyolysis-0  Metabolic acidosis-0 | Mortality-4/17  Morbidity- NA  MAP-NA  HR-NA  UO- increased, p=0.02  Catecholamine requirement-decreased, p<0.00001 | IV continuous AVP: 0.00005-0.001 units/kg/minute | 17 neonates | 1 day- 28 day | Hypotension (systemic inflammatory response, post cardiac surgery) | Observational-retrospective | Lechner et al, 2007[46] |
| Oxford-4 | 2 years | New onset tachycardia-0  Tissue ischemia/skin lesions-3  Cardiac arrest-0  Rhabdomyolysis-0  Metabolic acidosis-0 | Mortality-11/29  Morbidity- NA  MAP- increase, p=0.004  HR- decrease, p=0.014  UO- increase, p=0.001  Catecholamine requirement- decrease, p=0<0.001 | IV TP intermittent infusion: 7-20 mcg/kg/dose, up to 24 doses a day | 29 children (8 included in previous reports) | 2 days – 12 years | Vasodilatory (post cardiac surgery) | Observational-retrospective | Matok et al, 2009[47] |
| Oxford-4 | 1.2 years | New onset tachycardia-NA  Tissue ischemia/skin lesions-NA  Cardiac arrest-NA  Rhabdomyolysis-NA  Metabolic acidosis-NA | Mortality-2/11  Morbidity-see Web Table 2.  MAP-NA  HR- unchanged, p-NS  UO- unchanged, p=NS  Catecholamine requirement- decreased, p<0.005 | IV AVP continuous infusion: 0.0003-0.002 units/kg/minute | 11 children | 3 days – 15 years | Vasodilatory/ depressed cardiac function (post cardiac surgery) | Observational-retrospective | Rosenzweig et al, 1999[48] |
| Oxford- 4 | 32 months | New onset tachycardia-0  Tissue ischemia/skin lesions- 4  Cardiac arrest- 1  Rhabdomyolysis- 4  Metabolic acidosis- 3 | Mortality- 7/15  Morbidity- NA  MAP- increase, p<0.05  HR-decreased, p<0.05  UO-NA  Catecholamine requirement- decreased, p<0.01 | IV TP  loading dose: 20 mcg/kg  continuous infusion: 4-20 mcg/kg/hour | 15 children | 24 days-15 years | Septic | Case series | Rodriguez-Nunez et al, 2010[31] |
| Oxford-4 | 42 and 69 days | New onset tachycardia-0  Tissue ischemia/skin lesions-0  Cardiac arrest-0  Rhabdomyolysis-0  Metabolic acidosis-0 | Mortality-1/2  Morbidity- Web Table 2.  MAP- increase  HR-NA  UO- increase  Catecholamine requirement-NA | IV AVP continuous infusion: 0.0005-0.0011 units/kg/minute | 2 children | 2 years and 16 years | Septic | Case series | Inui et al, 2008[49] |
| Oxford-4 | 2 days- 15 days | New onset tachycardia-0  Tissue ischemia/skin lesions-0  Cardiac arrest-  Rhabdomyolysis-0  Metabolic acidosis-0 | Mortality-1/3  Morbidity- NA  MAP-NA  HR-NA  UO-NA  Catecholamine requirement-decreased | IV AVP continuous infusion: 0.0002-0.002 units/kg/minute | 3 children | 3 months- 13 years | Septic | Case series | Vasudevan,2005[50] |
| Oxford-I | 92 hours | New onset tachycardia-NA  Tissue ischemia/skin lesions-NA  Cardiac arrest-NA  Rhabdomyolysis-NA  Metabolic acidosis-NA | Mortality-1/2  Morbidity-NA  MAP-NA  HR-NA  UO-NA  Catecholamine requirement-1-decrease, 2-decrease | IV AVP continuous infusion: 0.0025-0.006 units/kg/minute | 2 adolescents | 12 and 15 years | Drug induced cardiac arrest/hypotension | Case series | Tobias et al, 2002[51] |
| Oxford-I | 1 week | New onset tachycardia-0  Tissue ischemia/skin lesions-0  Cardiac arrest-0  Rhabdomyolysis-0  Metabolic acidosis-0 | Mortality-0/1  Morbidity-NA  MAP-increase  HR-NA  UO-increase  Catecholamine requirement-NA | IV AVP continuous infusion:  0.0003 units/kg/minute | 1 child | 5 years | Septic | Case report | Vitale et al, 2011[52] |
| Oxford-I | 24 hours | New onset tachycardia-NA  Tissue ischemia/skin lesions-NA  Cardiac arrest-NA  Rhabdomyolysis-NA  Metabolic acidosis-NA | Mortality-0/1  Morbidity-NA  MAP-unchanged  HR-NA  UO-NA  Catecholamine requirement-decreased | IV TP bolus: 2 doses of 0.5mg | 1 child | 11 years | Septic | Case report | Peters, 2004[53] |
| Oxford-I | 18 hours | New onset tachycardia-0  Tissue ischemia/skin lesions-1  Cardiac arrest-  Rhabdomyolysis-0  Metabolic acidosis-0 | Mortality-1/1  Morbidity-NA  MAP-increase  HR-decrease  UO-NA  Catecholamine requirement-decrease | IV TP continuous infusion: 0.16 mcg/kg/minute | 1 child | 2 months | Septic | Case report | Zeballos, 2005[54] |
| Oxford-I | 14 days | New onset tachycardia-NA  Tissue ischemia/skin lesions-NA  Cardiac arrest-NA  Rhabdomyolysis-NA  Metabolic acidosis-NA | Mortality-1/1  Morbidity-NA  MAP-NA  HR-unchanged  UO-increase  Catecholamine requirement-NA | IV TP bolus: 7mcg/kg/dose, twice daily | 1 child | 8 days | Septic | Case report | Matok, 2004[55] |
| Oxford-I | 11 days | New onset tachycardia-0  Tissue ischemia/skin lesions-1  Cardiac arrest-0  Rhabdomyolysis-0  Metabolic acidosis-0 | Mortality-0/1  Morbidity-see Web Table 2.  MAP- increase  HR- unchanged  UO- increased  Catecholamine requirement- decreased | IV TP continuous infusion: 0.043 mcg/kg/minute | 1 child | 3 years | Septic | Case report | Michel et al, 2007[56] |
| Oxford-I | 19 days | New onset tachycardia-NA  Tissue ischemia/skin lesions-NA  Cardiac arrest-NA  Rhabdomyolysis-NA  Metabolic acidosis-NA | Mortality-1/1  Morbidity-NA  MAP-increase  HR-NA  UO-increase  Catecholamine requirement-decrease | IV TP bolus: 0.02mg/kg/dose, every 4 hours | 1 child | 13 days | Vasodilatory-septic | Case report | Filippi et al, 2008[57] |
| Oxford-I | 7 days | New onset tachycardia-NA  Tissue ischemia/skin lesions-NA  Cardiac arrest-NA  Rhabdomyolysis-NA  Metabolic acidosis-NA | Mortality-0/1  Morbidity- NA  MAP-NA  HR-NA  UO-NA  Catecholamine requirement- decrease | IV AVP:  Bolus: 5 Units continuous infusion: 0.0000005 -0.00003 units/kg/minute | 1 child | 11 years | Hypotension ( post-operative) | Case report | Deutsch et al, 2006[58] |
| Oxford-I | 2 days | New onset tachycardia-NA  Tissue ischemia/skin lesions-NA  Cardiac arrest-NA  Rhabdomyolysis-NA  Metabolic acidosis-NA | Mortality-0/1  Morbidity- see Web Table 2.  MAP-increase  HR-unchanged  UO-NA  Catecholamine requirement-NA | IV AVP continuous infusion: 0.00002-0.0003 units/kg/minute | 1 child | 13 years | Vasodilatory- (post cardiac surgey) | Case report | Lechner et al, 2004[59] |

* The above studies in WebTable 1.were not included in the meta-analysis *AVP-vasopressin, TP-terlipressin, MAP- mean arterial pressure (mmHg), HR- heart rate (beats/minute), UO- urine output, NA-not available- no report of the outcome/parameter/adverse events, I-irrelevant, VLBW/ELBW- very low/extremely low birth weight, Mortality-refers to PICU/NICU- pediatric/neonatal intensive care unit mortality.
