# Supplementary figures and images for: Role of vasopressin and terlipressin in refractory shock compared to conventional therapy in the neonatal and pediatric population: a systematic review, meta-analysis, and trial sequential analysis
Source: Crit Care. 2017 Jan 5;21:1. doi: 10.1186/s13054-016-1589-6 (PMC5217634; doi:10.1186/s13054-016-1589-6)

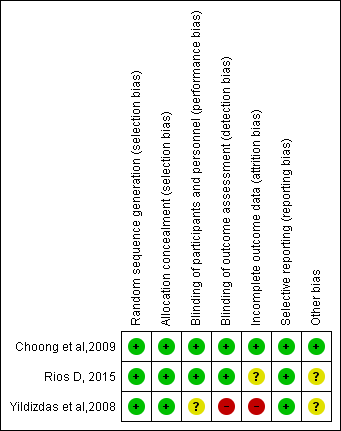

Supplement: Additional file 2: Figure S1. — Risk of bias summary: review authors' judgements about each risk of bias item for each included study. (DOCX 23 kb) [file 13054_2016_1589_MOESM2_ESM.docx]

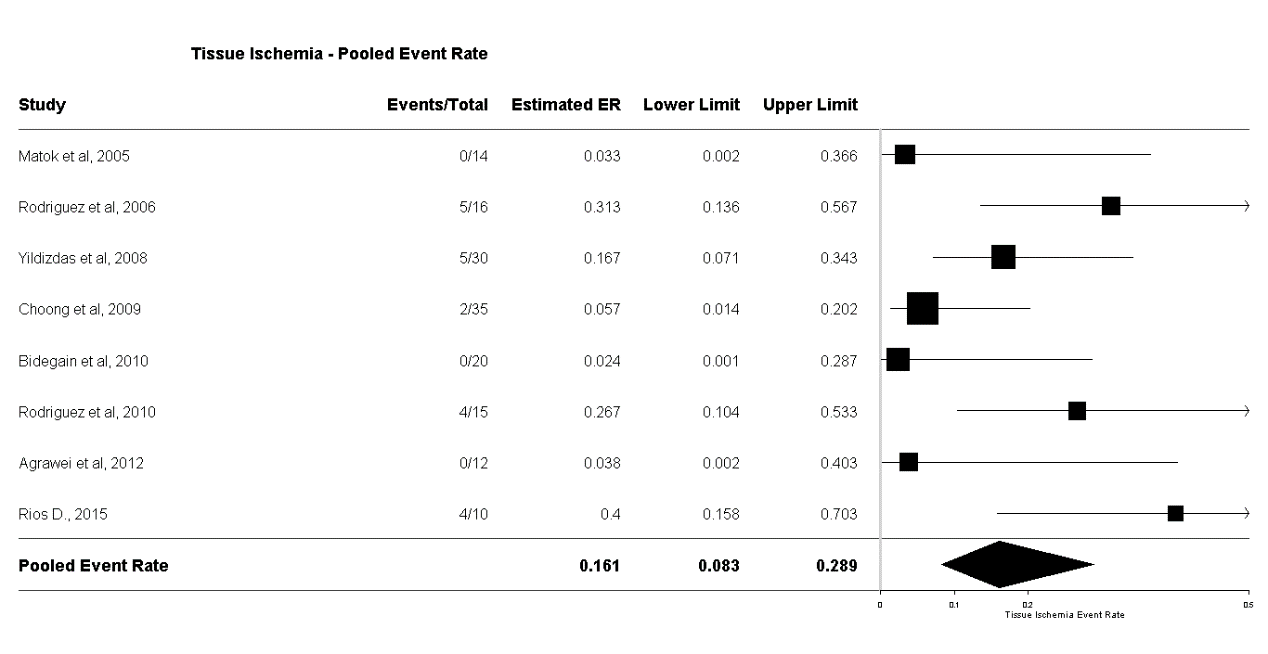

Supplement: Additional file 3: Figure S2. — Event rate for tissue ischemia in all clinical trials. The plot demonstrates point estimates of event rate surrounded by 95% CI. (DOCX 48 kb) [file 13054_2016_1589_MOESM3_ESM.docx]

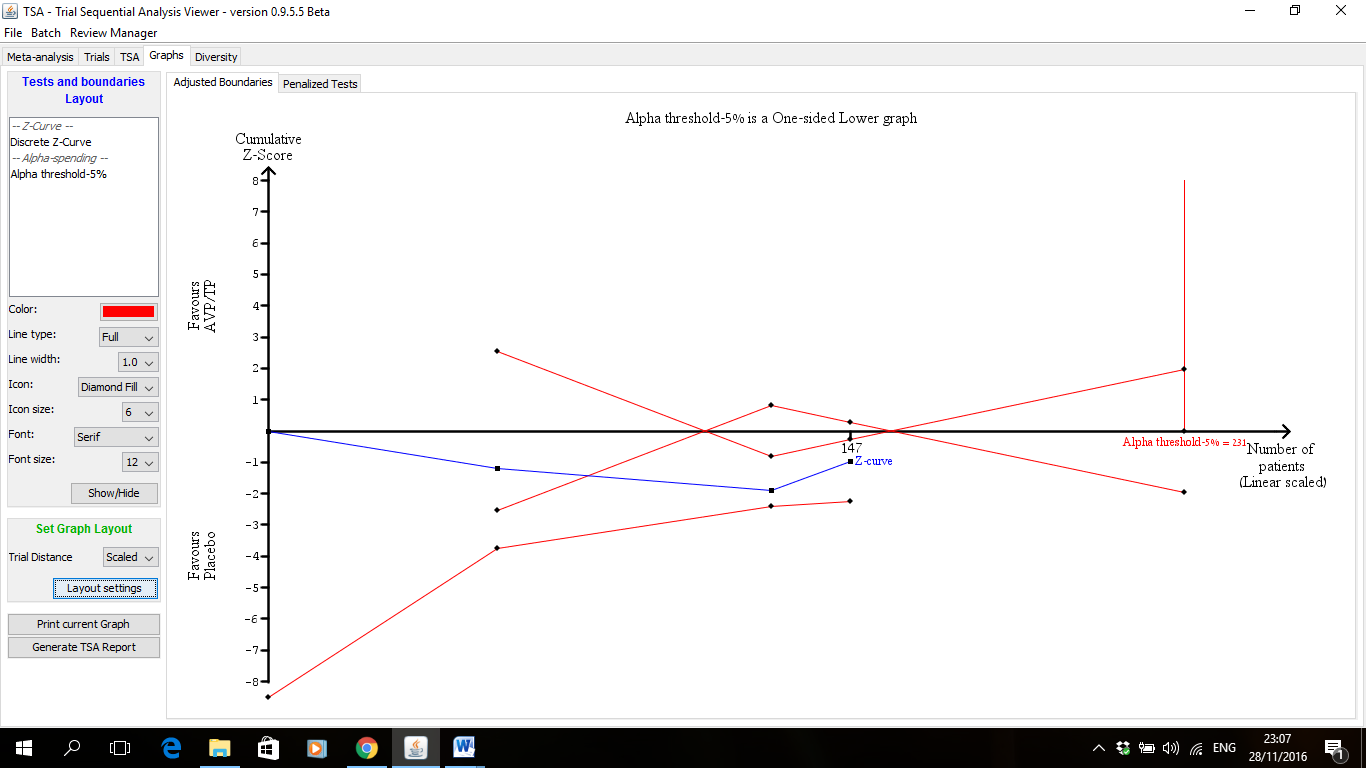

Supplement: Additional file 4: Figure S3. — Trial sequential analysis for tissue ischemia in randomized controlled trials. A relative risk of 2.72, one-sided lower boundary, an incidence of 1.6% in the control arm, an incidence of 11% in the treatment arm, a low bias estimated relative risk reduction of 85%, α of 5%, power of 80% were set. An estimated required information size of 231 randomized patients was not reached. The boundaries for futility are not crossed and no effect on tissue ischemia is observed. (DOCX 120 kb) [file 13054_2016_1589_MOESM4_ESM.docx]

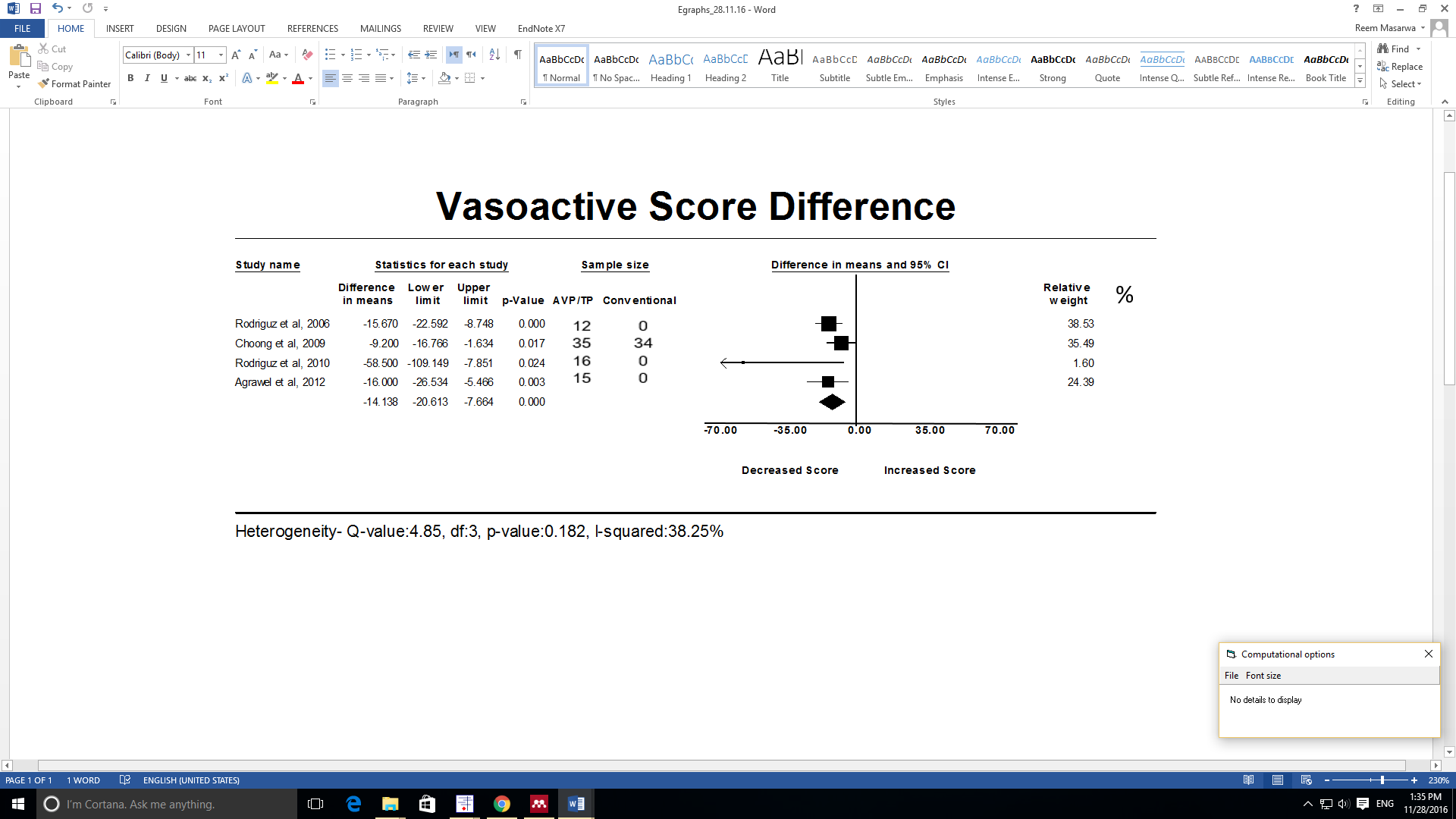


|  |  |
| --- | --- |
|  |  |
|  |  |
|  |  |
|  |  |

Supplement: Additional file 5: Figure S4. — Mean difference for vasoactive score. The forest plot demonstrates point estimates of mean difference surrounded by 95% CI. (DOCX 138 kb) [file 13054_2016_1589_MOESM5_ESM.docx]

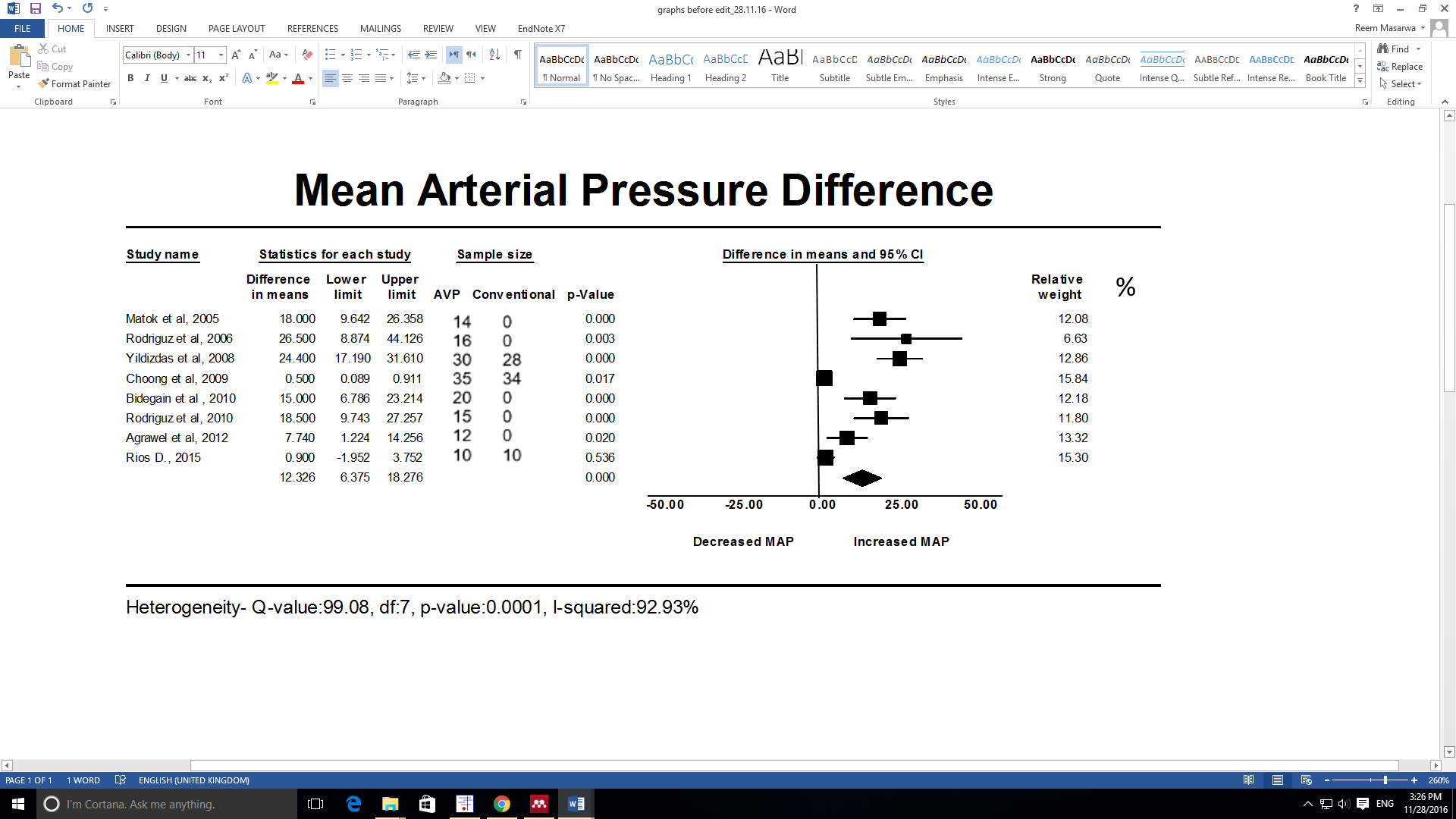


|  |  |
| --- | --- |
|  |  |
|  |  |
|  |  |
|  |  |
|  |  |
|  |  |
|  |  |

Supplement: Additional file 6: Figure S5. — Mean difference for mean arterial pressure. The forest plot demonstrates point estimates of mean difference surrounded by 95% CI. (DOCX 155 kb) [file 13054_2016_1589_MOESM6_ESM.docx]

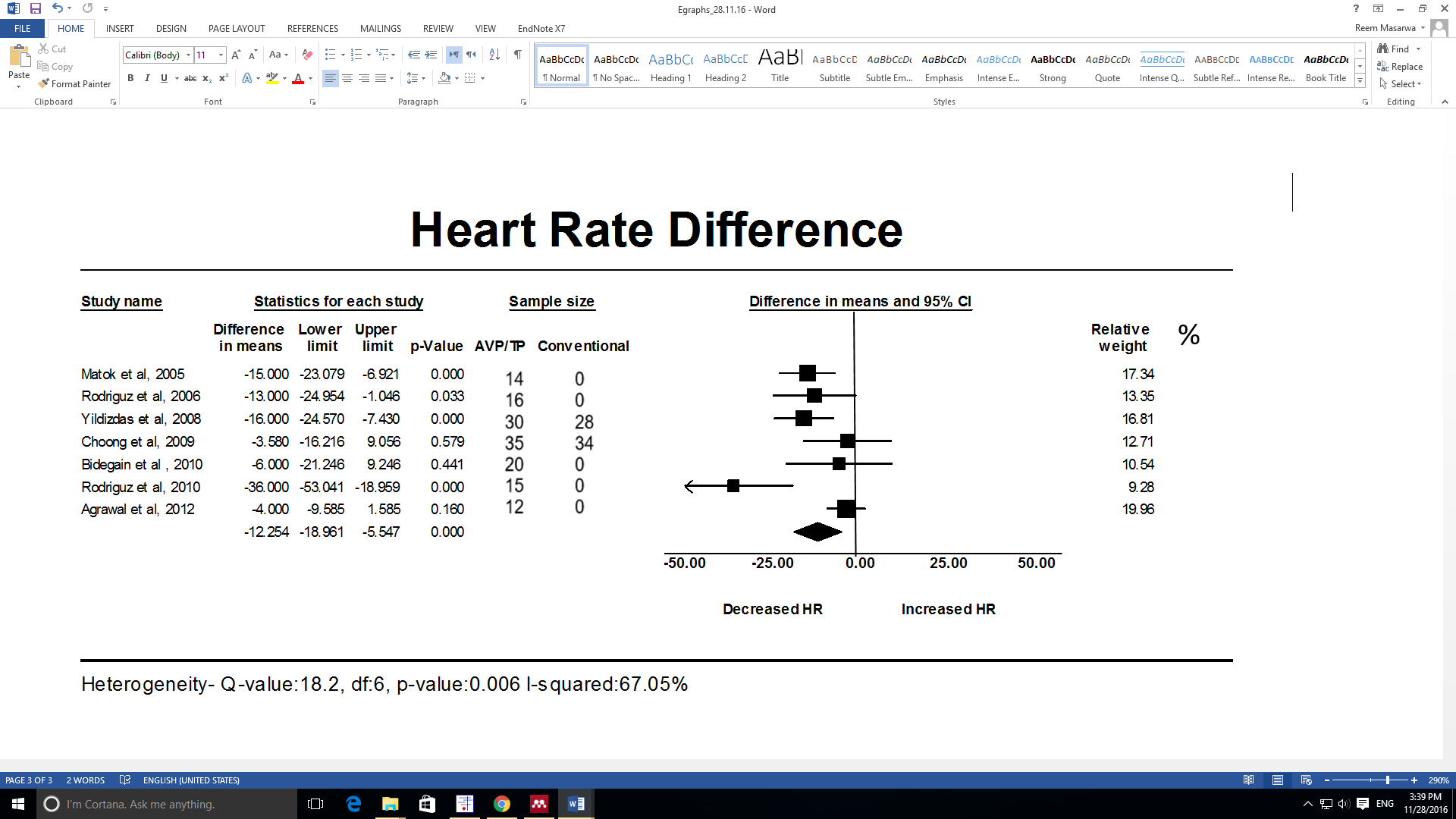


|  |  |
| --- | --- |
|  |  |
|  |  |
|  |  |
|  |  |
|  |  |
|  |  |

Supplement: Additional file 7: Figure S6. — Mean difference for heart rate. The forest plot demonstrates point estimates of mean difference surrounded by 95% CI. (DOCX 153 kb) [file 13054_2016_1589_MOESM7_ESM.docx]

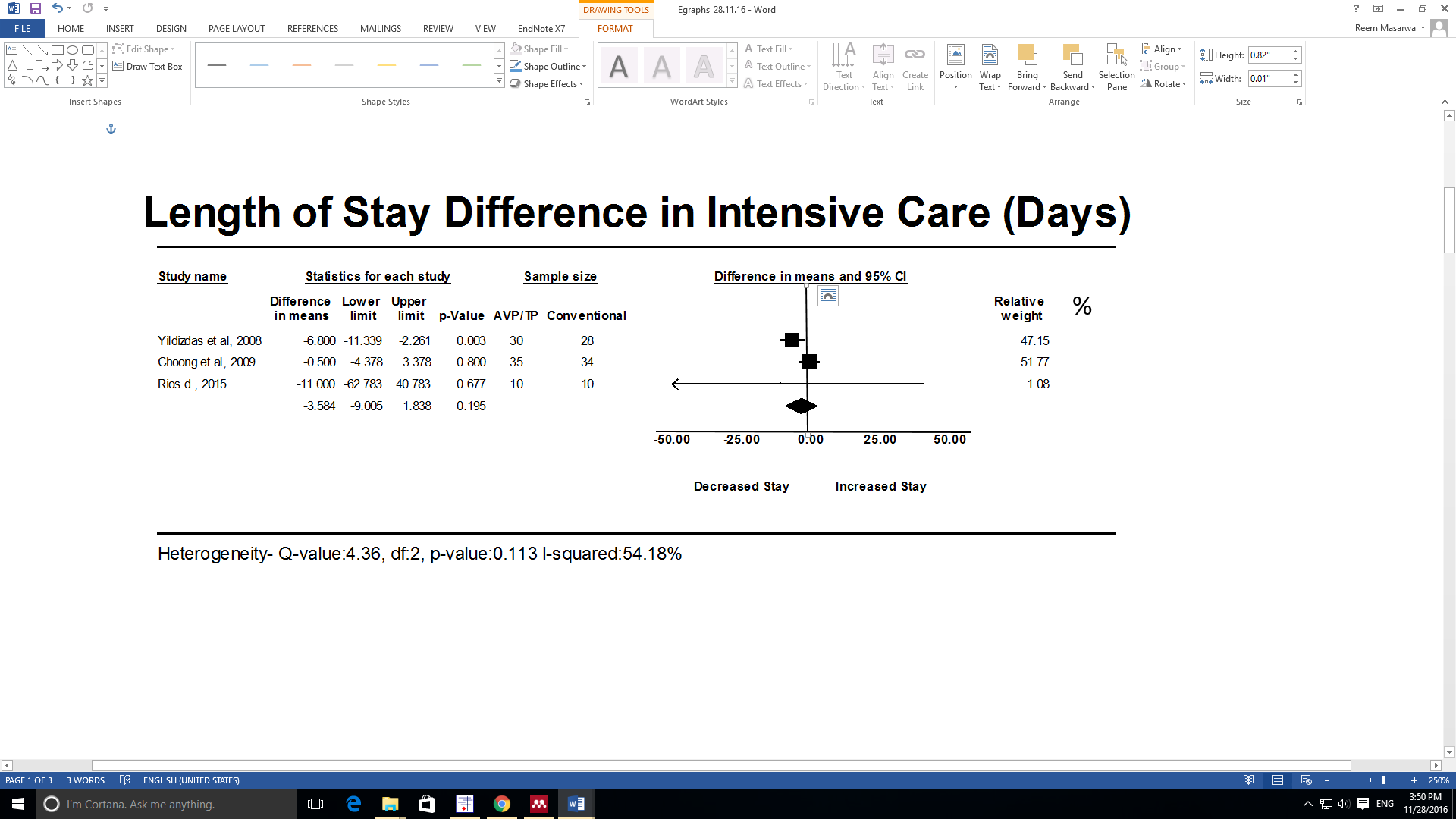

Supplement: Additional file 8: Figure S7. — Mean difference for length of stay in intensive care. The forest plot demonstrates point estimates of mean difference surrounded by 95% CI. (DOCX 133 kb) [file 13054_2016_1589_MOESM8_ESM.docx]
